# Supplementary material for: Assigning the Origin of Microbial Natural Products by Chemical Space Map and Machine Learning
Source: Biomolecules. 2020 Sep 28;10(10):1385. doi: 10.3390/biom10101385 (PMC7600738; doi:10.3390/biom10101385)
Supplement: Supplementary file 1 [file biomolecules-10-01385-s001.pdf]

Supplementary materials for:

Assigning the Origin of Microbial Natural Products by Chemical Space Map and Machine Learning

Alice Capecchi and Jean-Louis Reymond*

Department of Chemistry and Biochemistry, University of Bern, Freiestrasse 3, 3012 Bern, Switzerland

E-mail: jean-louis.reymond@dcb.unibe.ch

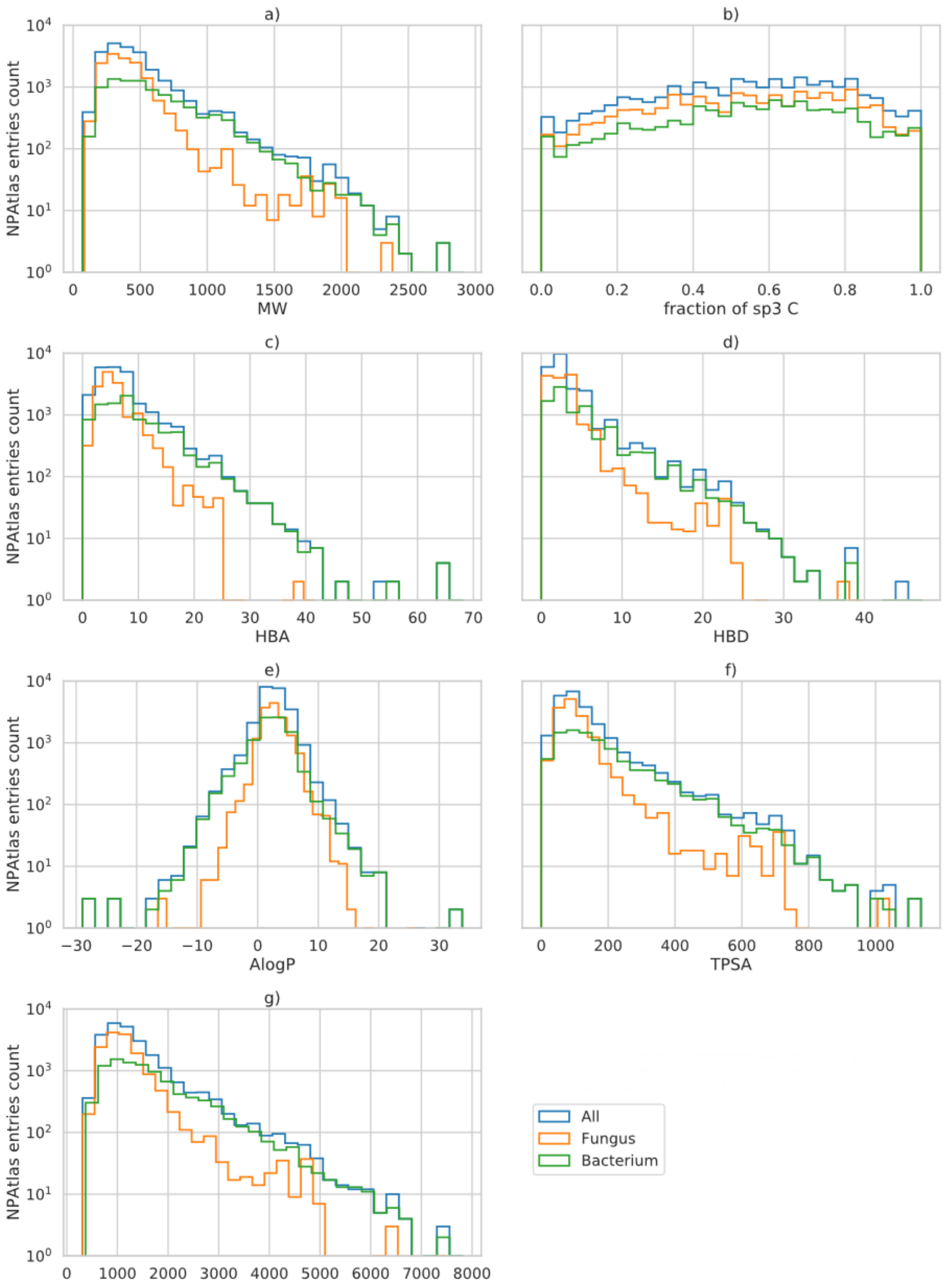

Figure S1. Physico-chemical properties distribution across the NPAtlas entries are shown in blue. The distribution within compounds of fungal and bacterial origin are also reported, in orange and green, respectively.

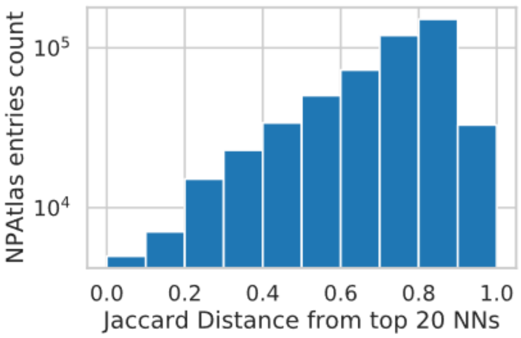

Figure S2. Approximated Jaccard distance from the top 20 NNs of all NPAtlas entries.

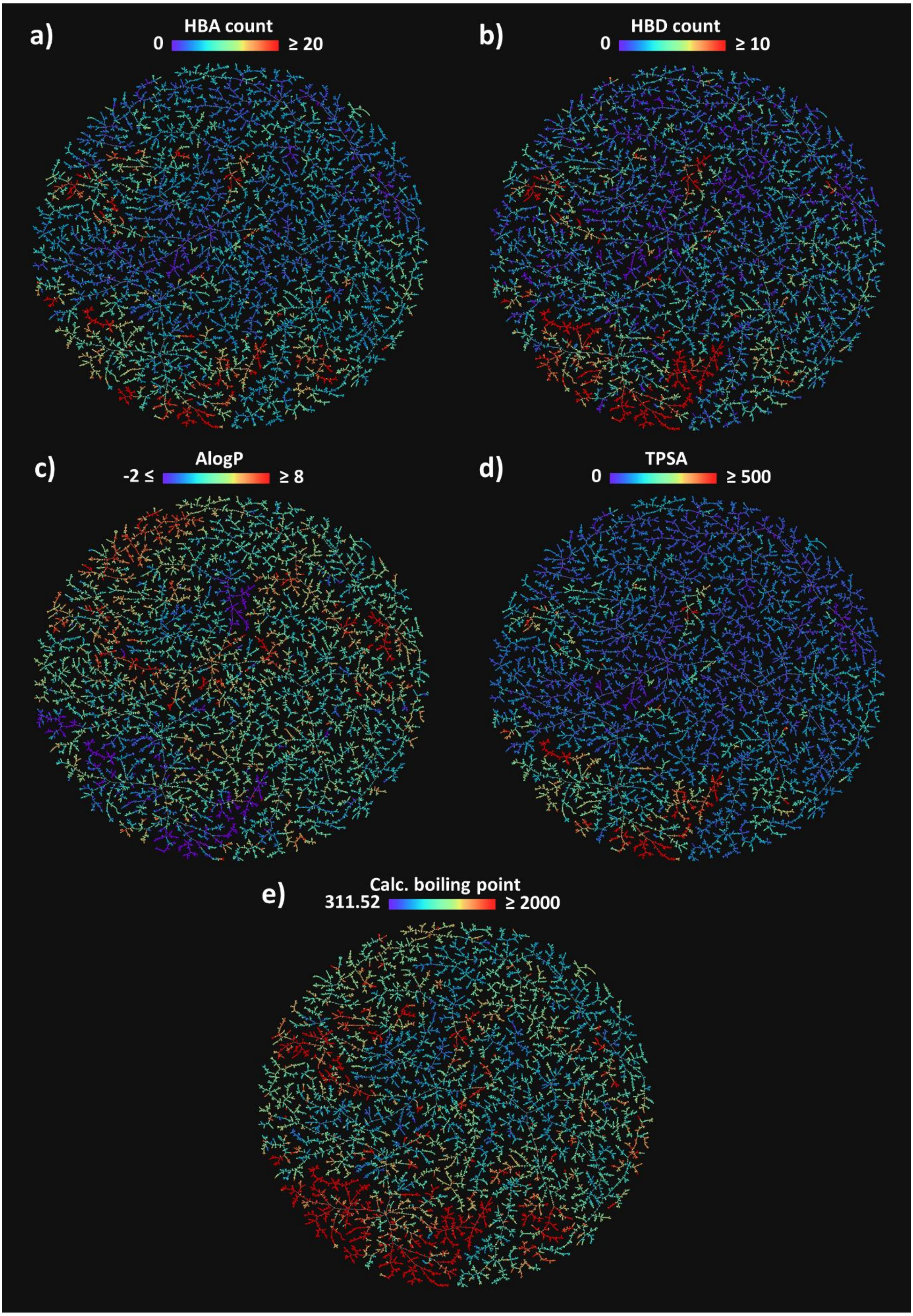

Figure S3. MAP4 TMAP of NPAtlas colored with the available continuous properties (a) HBA, (b) HBD, (c) AlogP, (d) TPSA, and (e) calculated boiling point.

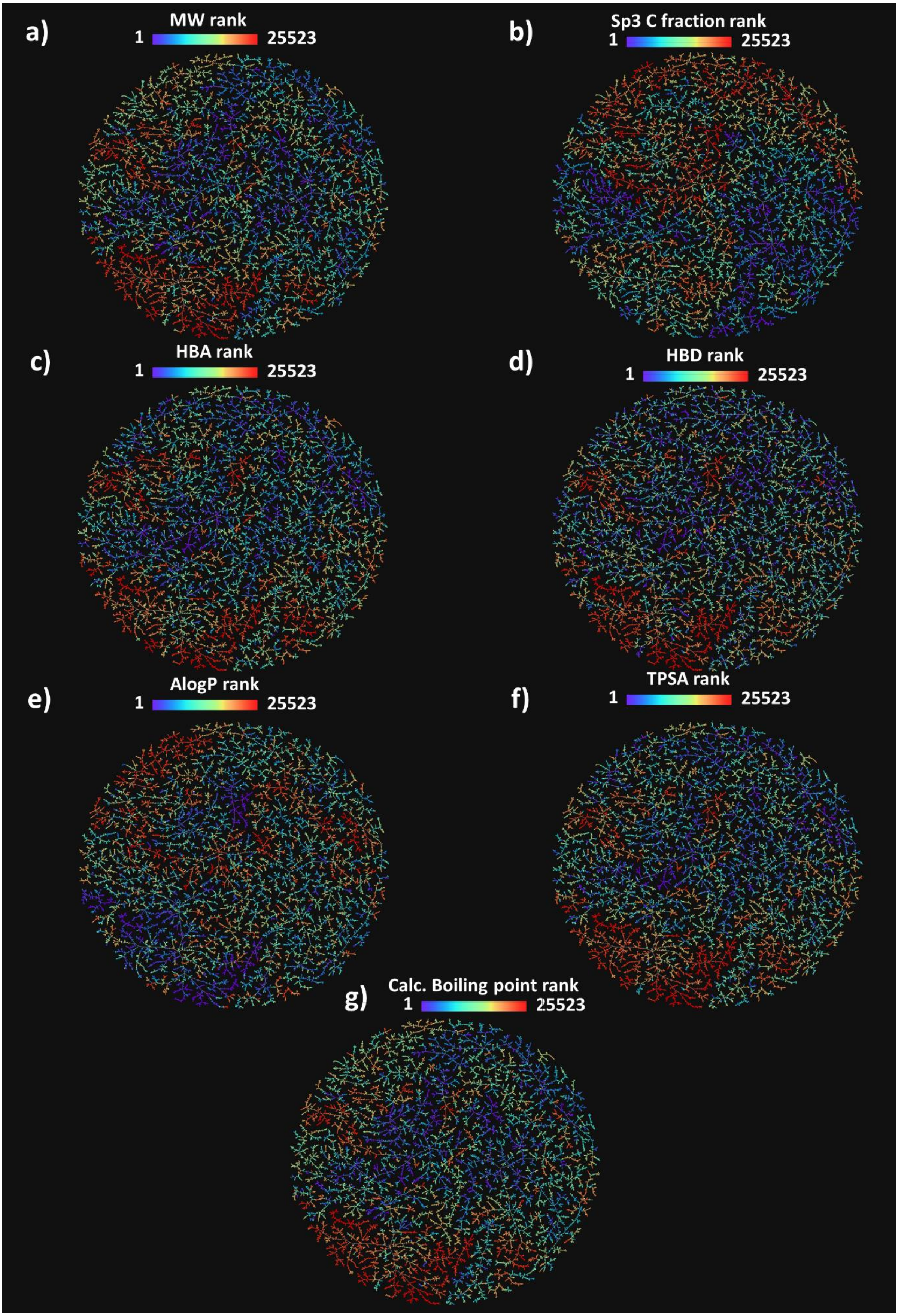

Figure S4. MAP4 TMAP of NPAtlas colored with the ranked continuous properties (a) MW, (b) fsp3C, (c) HBA, (d) HBD, (e) AlogP, (f) TPSA, (g) and calculated boiling point.

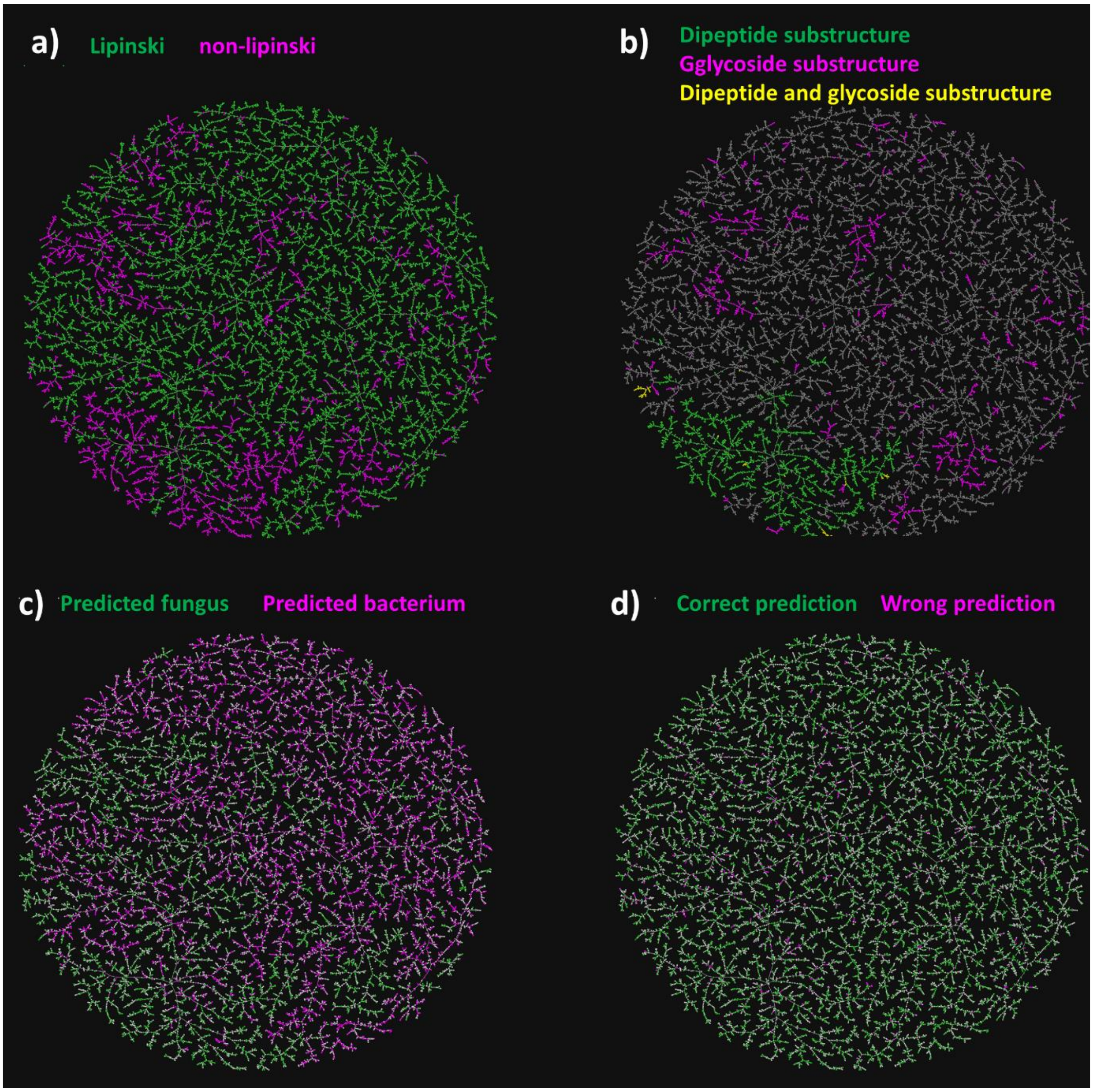

Figure S5. MAP4 TMAP of NPAtlas colored with categorical properties. (a) Lipinski classification. (b) Presence of glycoside and/or dipeptide substructures. (c) MAP4 SMV prediction. (d) MAP4 SVM performance.

Table S1. MAP4 SVMN classification of animal or plant NPs.

Natural Product	Origin[a)]	MAP4 SVM pred.[b)] fungal, bacterial	Training set NN	JD from NN[c)]
Salidroside	Plant	**0.75**, 0.25	NPA016219	0.75
Prostacyclin	Animal	**0.94**, 0.06	Shorghumoic acid (NPA005601)	0.78
Serricorole	Animal	0.16, **0.84**	6-deoxyerythronolide B (NPA004018)	0.81
Cholesterol	Animal	**0.93**, 0.07	Micaceol (NPA018196)	0.37
Farnesol	Plant, animal	**0.92**, 0.08	Trans-beta-Farnesene (NPA013150)	0.64
Menthol	Plant	**0.96**, 0.04	(+)-7-Hydroxymenthol (NPA012557)	0.50
Conotoxin MVIIA	Animal	0.13, **0.87**	Siamycin II (NPA020589)	0.75

a) Natural product origin. b) Predicted origin: fungal or bacterial. c) Approximated Jaccard Distance (JD, see methods for details) from the training set NN.

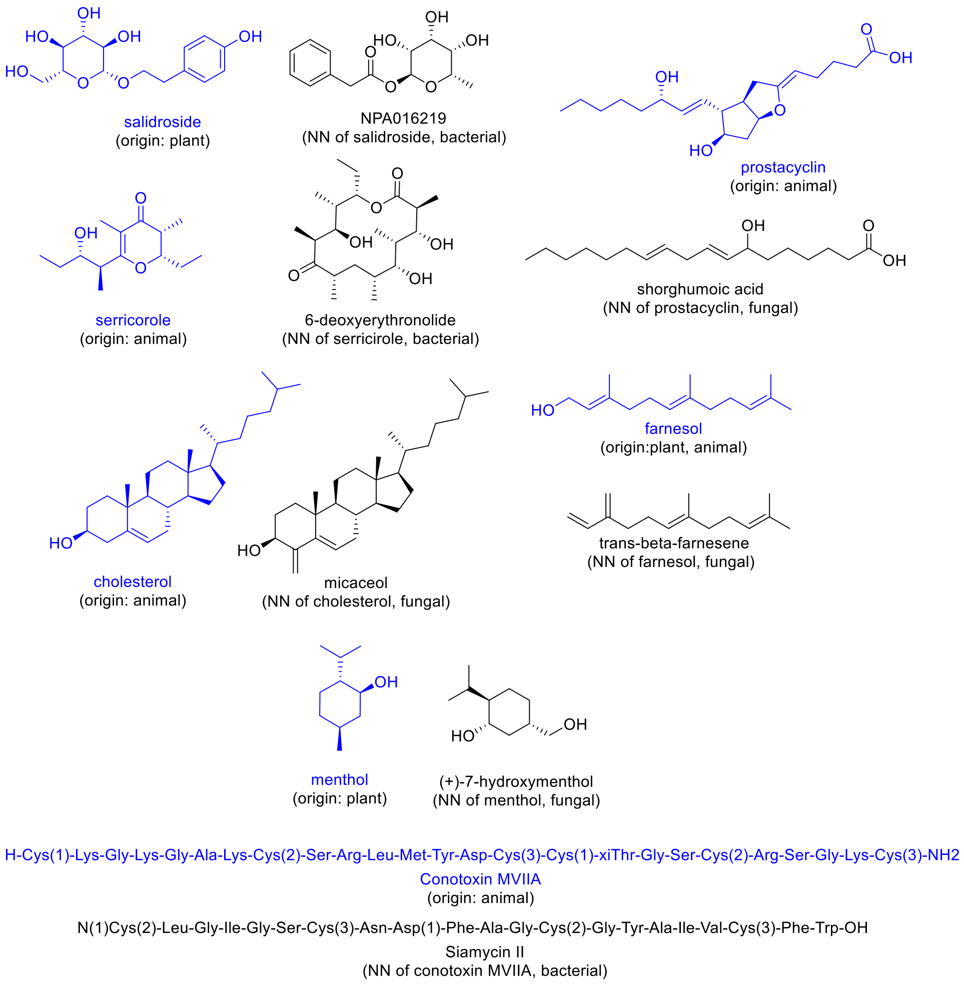

Figure S6. Examples of NPs from animals or plants (blue) and their NPAtlas training set NN (black). See also Table S1.
